# Validation of Surrogate Anthropometric Indices in Older Adults: What Is the Best Indicator of High Cardiometabolic Risk Factor Clustering?

**DOI:** 10.3390/nu11081701

**Published:** 2019-07-24

**Authors:** Robinson Ramírez-Vélez, Miguel Ángel Pérez-Sousa, Mikel Izquierdo, Carlos A. Cano-Gutierrez, Emilio González-Jiménez, Jacqueline Schmidt-RioValle, Katherine González-Ruíz, María Correa-Rodríguez

**Affiliations:** 1 Department of Health Sciences, Public University of Navarra, Navarrabiomed-Biomedical Research Centre, IDISNA-Navarra’s Health Research Institute, C/irunlarrea 3, Complejo Hospitalario de Navarra, 31008 Pamplona, Navarra, Spain; 2 Faculty of Sport Sciences, University of Huelva, Avenida de las Fuerzas Armadas s/n, 21007 Huelva, Spain; 3 Centro de Investigación Biomédica en Red de Fragilidad y Envejecimiento Saludable (CIBERFES), Instituto de Salud Carlos III, 28029 Madrid, Spain; 4 Hospital Universitario San Ignacio – Aging Institute, Pontificia Universidad Javeriana, Bogotá 110111, Colombia; 5 Department of Nursing, Faculty of Health Sciences, University of Granada, Av. Ilustración, 60, 18016 Granada, Spain; 6 Grupo de Ejercicio Físico y Deportes, Vicerrectoría de Investigaciones, Facultad de Salud, Universidad Manuela Beltrán, Bogotá 110231, DC, Colombia

**Keywords:** anthropometric indices, diagnosis criteria, metabolic syndrome, cardiometabolic risk, elderly

## Abstract

The present study evaluated the ability of five obesity-related parameters, including a body shape index (ABSI), conicity index (CI), body roundness index (BRI), body mass index (BMI), and waist-to-height ratio (WtHR) for predicting increased cardiometabolic risk in a population of elderly Colombians. A cross-sectional study was conducted on 1502 participants (60.3% women, mean age 70 ± 7.6 years) and subjects’ weight, height, waist circumference, serum lipid indices, blood pressure, and fasting plasma glucose were measured. A cardiometabolic risk index (CMRI) was calculated using the participants’ systolic and diastolic blood pressure, triglycerides, high-density lipoprotein and fasting glucose levels, and waist circumference. Following the International Diabetes Federation definition, metabolic syndrome was defined as having three or more metabolic abnormalities. All surrogate anthropometric indices correlated significantly with CMRI (*p* < 0.01). Receiver operating characteristic curve analysis of how well the anthropometric indices identified high cardiometabolic risk showed that WtHR and BRI were the most accurate indices. The best WtHR and BRI cut-off points in men were 0.56 (area under curve, AUC 0.77) and 4.71 (AUC 0.77), respectively. For women, the WtHR and BRI cut-off points were 0.63 (AUC 0.77) and 6.20 (AUC 0.77), respectively. In conclusion, BRI and WtHR have a moderate discriminating power for detecting high cardiometabolic risk in older Colombian adults, supporting the idea that both anthropometric indices are useful screening tools for use in the elderly.

## 1. Introduction

Metabolic syndrome (MetS) is a complex cluster of cardiovascular risk factors associated with a sedentary lifestyle, poor nutrition, and consequent overweight. It is also strongly associated with other abnormalities linked to cardiovascular disease (CVD), including glucose intolerance (type 2 diabetes, impaired glucose tolerance, or impaired fasting glycemia), insulin resistance, abdominal obesity, dyslipidemia, and hypertension [1]. Accordingly, MetS increases the risk of developing diseases of cardiovascular origin, such as acute myocardial infarction, ischemic stroke, or coronary heart disease [2]. Indeed, the prevalence of CVD attributable to MetS is estimated at around 12–17% [3]. Several studies have examined the presence of MetS in Latin America, reporting associated factors including advanced age, having Hispanic or indigenous heritage, physical inactivity, high alcohol intake, smoking, history of hypertension or type 2 diabetes (first-degree family members), and having a low socioeconomic status (reviewed in [4]). The general prevalence of MetS in Latin-American countries has been established as 24.9% (range: 18.8–43.3%) and is slightly more frequent in women (25.3%) than in men (23.2%).

The clinical utility of identifying MetS in older adults has been much debated because, among the issues raised, it has been argued that there is no consensus on the clinical criteria for screening the elderly population to identify patients likely to be characterized with MetS. In this line, several clinical criteria and cut-off points have been proposed. For instance, the cardiometabolic risk index (CMRI) in older adults, measured as a continuous summary score, might represent an important intermediate or preclinical outcome that can be measured prior to the onset of disease, and could provide opportunities for prevention. As a marker of cardiometabolic disease risk, the use of adult CMRI severity z-scores has been suggested as an accurate method to detect overall metabolic changes [5]. This continuous score would be more sensitive to small and large changes that do not modify the most recent Joint Interim Statement of the International Diabetes Federation (IDF) Task Force on Epidemiology and Prevention criteria [6]. Thus, an increase in cholesterol from 150 to 250 mg/dl would have no impact on the IDF score, but would be reflected as a non-trivial change in the continuous CMRI [7]. Nevertheless, there is no validated or harmonized consensus for defining CMRI in older adults, and several continuous CMRI scores have been reported in the literature, as described in previous narrative reviews.

Measurements of anthropometric indices are inexpensive and non-invasive, and are easily conducted as part of normal health exams. Interestingly, anthropometric measurements such as body mass index (BMI), waist circumference (WC), and waist-to-height ratio (WtHR) show a close correlation with MetS components and could thus be useful surrogate markers for predicting MetS [8,9,10]. That being said, there remains controversy over which anthropometric indices [11] are the most appropriate predictors of cardiometabolic disease [12]. In 2012, Krakauer and Krakauer developed “A Body Shape Index” (ABSI), based on WC adjusted for height and weight [13], and demonstrated that a high ABSI is associated with the accumulation of excess abdominal adipose tissue and seems to be a substantial risk factor for premature mortality in the general population [13]. In a similar vein, the conicity index (CI), an index of abdominal obesity, has been considered useful for detecting central obesity, and has been studied as a predictor for alterations in fasting insulin, blood pressure, and triglyceride levels [14]. Lastly, in 2013, Thomas and colleagues [15] developed the body roundness index (BRI), which combines height and WC to predict the percentage of body fat. When compared with other anthropometric indices, BRI was optimal for identifying MetS, insulin resistance, inflammatory factors [16], and arterial stiffness [17] in obese and overweight populations. However, to date, few studies have evaluated the predictive ability of BRI, ABSI, or CI compared with traditional metrics, such as BMI and WtHR, with regard to CMRI in older adults [18,19,20].

South America has undergone a rapid epidemiologic transition, including a non-communicable disease epidemic [21] and adverse lifestyle changes that could contribute to increase a cluster of cardiometabolic risk factors such as MetS [4]. To the best of our knowledge, the predictive power of anthropometric measurements, which can be measured easily in a routine health exam, has not been assessed in elderly Latin-American individuals with high cardiovascular risk, for whom the early detection of risk factors is essential for prevention of CVD. This is particularly true in Colombia, where anthropometric index measurements and blood collection are not usually standard in the annual health exam, and, to date, there have been few studies conducted in the general older population.

For these reasons, the aim of the present study was to evaluate the prevalence of MetS using a CMRI among older adults from Colombia, and validate the associated anthropometric surrogate markers. We also compared the predictive ability of BRI, ABSI, CI, BMI, and WtHR to determine whether there is a single best CMRI predictor.

## 2. Materials and Methods

### 2.1. Study Design and Participants

The data for this secondary cross-sectional study was obtained from the 2015 Colombian Health, Well-Being and Aging Survey (SABE 2015, from the Spanish: SAlud, Bienestar and Envejecimiento, 2015), a multicenter project conducted from 2014 to 2015 by the Pan-American Health Organization and supported by the Epidemiological Office of the Ministry of Health and Social Protection of Colombia (https://www.minsalud.gov.co/). The survey is a cross-sectional tool for exploring and evaluating several aspects that intervene in the phenomenon of aging and old age in the Colombian population [19]. Details of the survey have been previously published [19]. SABE 2015 was a joint venture between the Ministry of Health and Social Protection and the Administrative Department of Science, Technology and Innovation in Colombia.

The sample was regionally representative and involved self-representation in large cities, with urban-rural stratification of the sample and stage selection in accordance with the municipal map available from the Ministry of Health and Social Protection, with the following hierarchy: municipalities, urban/rural segments, homes or sidewalks, homes, and people. The study included the Colombian population ≥60 years old, and the indicators were disaggregated by age range, sex, ethnicity, and socioeconomic level. To calculate the original sample size, the non-institutionalized Colombian population aged ≥60 years was considered, and the following parameters were used: minimum estimable proportion = 0.03, design effect = 1.2, and Relative Standard Error = 0.05 (1.2). The universe of study comprised 99% of the population residing in private homes in both urban and rural areas.

A total of 23,694 surveys were conducted across the country and 6365 total population segments were investigated in 246 municipalities. As Bogotá is the capital it was independently selected, with a total of 545 urban segments and one rural segment. The average number of adults per segment was 4.2. The estimation of means or proportions was conducted to a level of precision of up to 6% of the maximum expected error, at a level of national disaggregation only. The basic procedure for the population survey was a face-to-face interview using a structured questionnaire. The interviewers visited the selected homes, carrying the appropriate identification. At each home visited, the standardized process involved the following: identifying the participants, registering the demographic data, obtaining the signed informed consent, applying the established filters and selection criteria, obtaining a signed assent form when necessary, and completion of the questionnaire by the interviewer. A total of 1502 participants from 86 municipalities were included in this analysis.

The institutional review boards involved in developing the SABE 2015 study (the University of Caldas, ID protocol CBCS-021-14, and the University of Valle, ID protocol 09-014 and O11-015) reviewed and approved the study protocol. Written informed consent was obtained from each individual before inclusion and completion of the first examination. One of the authors (C.A.C.-G.) applied to the Ministry of Health and Social Protection of Colombia and obtained permission to use publicly available data for research and teaching purposes (permission and details available at https://www.minsalud.gov.co/). The study protocol for the secondary analysis was approved by the Human Subjects Committee at the Pontificia Universidad Javeriana (ID protocol 20/2017-2017/180, FM-CIE-0459-17) in accordance with the Declaration of Helsinki (World Medical Association) and Resolution 8430 from 1993, of the then Colombian Ministry of Health, on technical, scientific, and administrative standards for conducting research with humans.

### 2.2. Anthropometric Measurements

The research teams of the coordinating centers (Caldas and Valle universities, Colombia) trained the data collection staff to carry out the face-to-face interviews and physical measurements. Anthropometric measurements included height and body weight, which were measured using a portable stadiometer (SECA 213^®^, Hamburg, Germany) and an electronic scale (Kendall graduated platform scale), respectively. BMI was estimated in kg/m^2^ from the measured body weight and height. WC was measured using inextensible anthropometric tape with the subjects standing erect and relaxed, with their arms at their sides and their feet positioned close together, parallel to the floor. WtHR was calculated as the ratio of WC (cm) to height (cm). The other anthropometric indexes (BRI, ABSI, and CI) were calculated using the following formulas: BRI = 364.2 − 365.5 (1 − π-^2^ WC^2^ (m) Height^−2^ (m))^1/2^ [15]; ABSI = WC (m)/(BMI^2/3^(kg/m^2^)Height^1/2^ (m)) [13]; CI = 0.109^−^^1^ WC (m) (Weight (kg)/Height (m))*^−^*^1/2^ [22].

### 2.3. Serum Biochemical Examination

After an overnight fast, blood was collected in the morning. Blood samples were centrifuged for 10 min at 3000 rpm, 30 min after sampling. All samples were delivered to a single central laboratory (Dinamica Laboratories, Bogotá, Colombia) for analysis within 24 h. Serum fasting glucose, low-density lipoprotein cholesterol (LDL-C), high-density lipoprotein cholesterol (HDL-C), total cholesterol, and triglycerides (TG) were analyzed using enzymatic colorimetric methods (Olympus AU5200, Melville, NY, USA). Low-density lipoprotein cholesterol (LDL-C) was estimated using the Friedewald equation ((LDL-C) = (Total Cholesterol) – (HDL-C) − ((TG)/5)).

### 2.4. Blood Pressure Determination

We measured systolic (SBP) and diastolic (DBP) blood pressure levels using an automatic blood pressure monitor (OMRON HEM-705, Omron Healthcare Co., Ltd., Kyoto, Japan), following the recommendations of the American College of Cardiology Foundation/American Heart Association 2011 Expert Consensus Document on Hypertension in the Elderly [23]. Values were recorded after 5 min of rest in the sitting position and three consecutive measures were obtained, waiting for at least 30 s between readings. The average of the three values for each measurement were used in the analysis.

### 2.5. Diagnostic Criteria of Metabolic Syndrome

MetS was defined according to the most recent Joint Interim Statement of the IDF [6] by adopting the Ethnic Central and South American criteria for WC. Participants were classified as having MetS if they had at least three of following metabolic risk factors or components (MetS-components): abdominal obesity (WC  ≥90 cm for Latin-American males and ≥80 cm for Latin-American females), elevated TG (fasting serum TG  ≥150 mg/dL or taking medication for abnormal lipid levels), low HDL-C (fasting serum HDL-C <40 mg/dL in males and <50 mg/dL in females, or specific treatment for this lipid abnormality), elevated blood pressure (SBP ≥130 mmHg or DBP ≥85 mmHg or taking hypertension medication), or elevated fasting glucose (serum glucose level ≥100 mg/dL or taking diabetes medication).

### 2.6. Definition of Cardiometabolic Risk Index

We calculated the CMRI as a continuous score of the MetS risk factors. The CMRI was calculated using sex- and race-specific algorithms for the IDF criteria cut-off values, using the values of the participants’ SBP and DBP, TG, HDL-C, fasting glucose, and WC. For each of these variables, a z-score was computed as the number of standard deviation (SD) units from the sample mean after normalization of the variables, that is, z-score = ((value − sample mean)/sample SD)). The HDL-C z-score was multiplied by −1 to indicate higher cardiovascular risk with increasing value. Individuals with a CMRI ≥ 1 SD above the mean were identified as having increased cardiometabolic risk, and a lower CMRI (<1 SD) being indicative of a healthier risk profile.

### 2.7. Co-Variables

For lifestyle characteristics, personal habits regarding alcohol intake (participants were categorized as those who do not drink and those who drink less than one day per week, two to six days a week, or every day) and cigarette smoking (participants were categorized as those who do not smoke and those who have never-smoked, those who currently smoke or those who previously smoked) were recorded. A “proxy physical activity” report was conducted by the following questions: (i) “Have you regularly exercised, such as jogging or dancing, or performed rigorous physical activity at least three times a week for the past year?”; (ii) “do you walk at least three times a week between nine and 20 blocks (1.6 km) without resting?”; (iii) “do you walk at least three times a week eight blocks (0.5 km) without resting?”. Participants were considered physically active if they responded affirmatively to two of the three questions [24].

Medical information including multimorbidity, as well as chronic conditions adapted from the original SABE study, was assessed by asking the participants if they had been medically diagnosed with hypertension, type 2 diabetes mellitus, chronic obstructive pulmonary disease, CVD (heart attack, angina), stroke, cancer, arthritis, osteoporosis, or sensory impairments (vision and hearing loss).

Race/ethnicity was self-reported and grouped into indigenous (people belonging to various indigenous groups such as Ika, Kankuamo, Emberá, Misak, Nasa, Wayuu, Awuá, and Mokane); black, “mulatto”, or Afro-Colombian; white; and other (mestizo, gypsy, etc.).

Socioeconomic status was determined on a scale of one to six based on the housing stratum, with one representing the highest level of poverty and six the greatest wealth. This classification was developed by the National Government of Colombia and considers the physical characteristics of the dwellings as well as their surroundings. Classification into one of the six strata was taken to approximate the hierarchical socioeconomic differences from poverty to wealth.

### 2.8. Statistical Analysis

Descriptive analyses using the mean ± SD or standard error (SE) for the continuous variables, median and interquartile range for the skewed continuous variables, and the frequency distribution of the categorical variables were used to determine the characteristics of the sample. Data normality was examined using the Kolmogorov–Smirnoff test. Significant differences between men and women were analyzed using Student’s *t*-test, Wilcoxon rank-sum test, or chi-square (χ^2^) post-hoc test. To visualize the relationship between CMRI and anthropometric indices, Spearman and Pearson correlation and linear regression analysis were applied to the total sample and individual genders. The linear regression analysis was adjusted by age as a covariate.

The area under receiver operating characteristic (ROC) curves was calculated to evaluate the abilities of the anthropometric indices to predict high CMRI. Cut-off points were proposed after calculation of Youden’s Index (sensitivity + specificity − 1) [25]. The DeLong et al. [26] non-parametric approach was used to compare the areas under the ROC curves. Since abdominal obesity is a component of CMRI, we conducted a multicollinearity test for the anthropometric indices that included WC (WtHR, CI, BRI, and ABSI), and the variance inflation factor (VIF) was calculated. Each cardiometabolic risk factor among BMI, WtHR, BRI, ABSI, and CI was determined using analysis of variance without any adjustment and then after adjusting (analysis of covariance, ANCOVA) for ethnicity, socio-economic status, smoking status, alcohol intake, physical activity “proxy”, and medical conditions (i.e., presence or absence of osteoporosis, CVD, hypertension, type 2 diabetes, cancer, or respiratory diseases) as covariates, followed by Tukey’s test. Collinearity was tested between all anthropometric indexes that included WC; a VFI > 10, was interpreted as high collinearity [27].

Statistical analyses were performed using SPSS v24.0 (IBM, Armonk, NY, USA) and JASP v0.9 (JASP Team, Amsterdam, The Netherlands). Statistical significance was defined as *p* < 0.05.

## 3. Results

### 3.1. Baseline Characteristics of the Participants

The participants´ characteristics are summarized in Table 1. Of the 1502 older adults studied, 60.3% were women, and the mean age was 70 ± 7.6 years. The prevalence of smoking (9.7%), alcohol intake (12.7%), and a physical activity proxy (17.7%) was relatively low, but significantly higher in CMRI ≥ 1 SD than in CMRI < 1 SD (alcohol: 13.1% vs. 12.6%, *p* < 0.001). The means (SD or range interquartile) of the WtHR, BMI, BRI, ABSI, and CI in the overall sample were 0.59 (0.1), 27.3 (24−30) kg/m^2^, 5.2 (4.1−6.3), 0.081 (0.078−0.085), and 22.2 (20.9−23.8), respectively. The overall prevalence of MetS was 58.7%. Significant differences were found between the high/low CMRI status groups for almost all characteristics, with the exception of height, LDL-C and HDL-C levels.

### 3.2. Association between Surrogate Anthropometric Indices with CMRI

**Figure 1:**
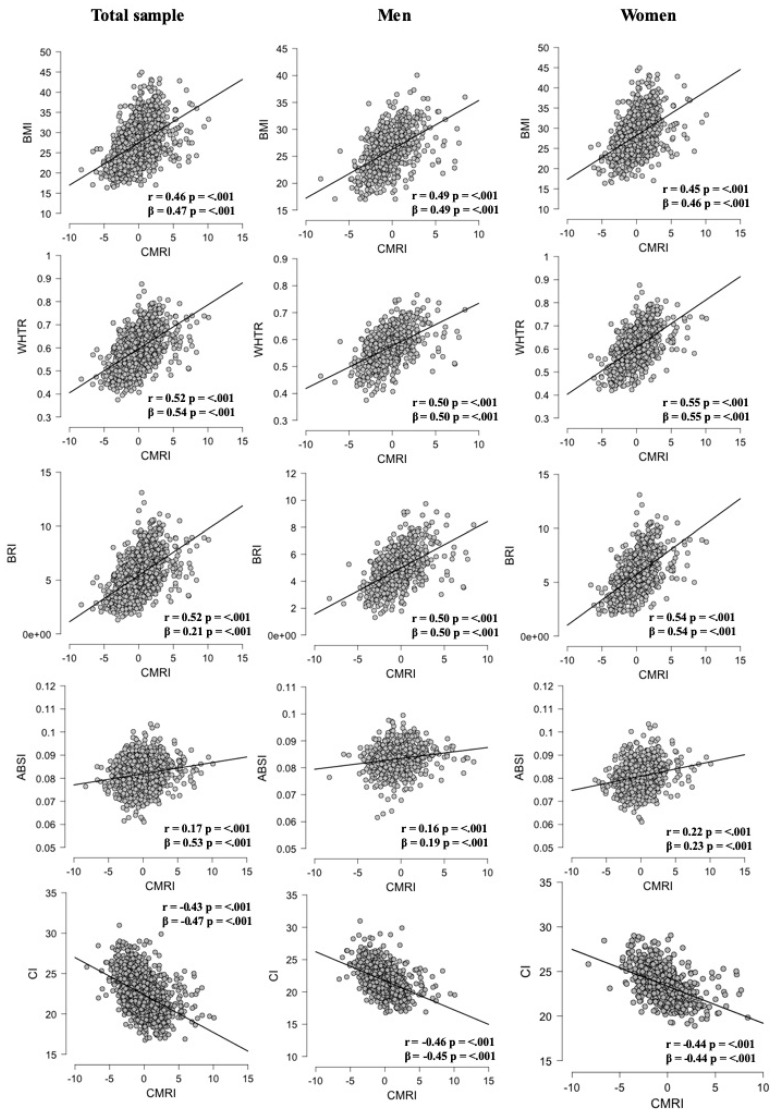
Association between surrogate anthropometric indices and CMRI, on the total sample and stratified by sex. BMI: body mass index; WtHR: waist to height ratio; BRI: body roundness index; ABSI: a body shape index; CI: conicity index; CMRI: cardiometabolic risk index.

Linear regression analyses of surrogate anthropometric indices and CMRI on the total sample and also stratified by sex are shown in Figure 1. Overall, we found an acceptable-to-moderate positive correlation of CMRI with WtHR (*r* = 0.52, *p* < 0.001), ABSI (*r* = 0.17, *p* < 0.001), BMI (*r* = 0.46, *p* < 0.001), and BRI (*r* = 0.52, *p* < 0.001), whereas CI was negatively correlated with CMRI (*r* = −0.42, *p* < 0.001). When analyzing by sex, the decreasing order of the correlation coefficients in men was WtHR (*r =* 0.50, *p* < 0.001), BRI (*r* = 0.50, *p* < 0.001), BMI (*r* = 0.49, *p* < 0.001), CI (*r* = −0.46, *p* < 0.001), and ABSI (*r* = 0.16, *p* < 0.001), while in women the decreasing order of the correlation coefficients was WtHR (*r* = 0.55, *p* < 0.001), BRI (*r* = 0.54, *p* < 0.001), BMI (*r* = 0.45, *p* < 0.01), CI (*r* = −0.44, *p* < 0.001), and ABSI (*r* = 0.22, *p* < 0.001).

### 3.3. Optimal Cut-Offs for Screening for CMRI by Sex

**Figure 2:**
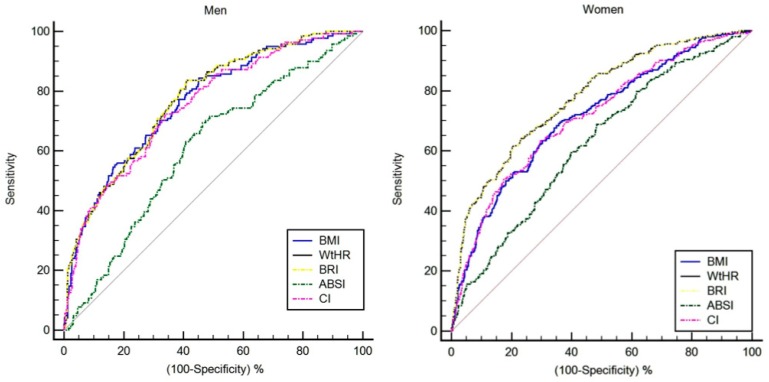
Diagnostic performance of surrogate anthropometric indices to detect high risk of CMRI by gender. BMI: body mass index; WtHR: waist-to-height ratio; BRI: body roundness index; ABSI: a body shape index; CI: conicity index.

The ROC curve analyses of the diagnostic performance of BMI, WtHR, BRI, ABSI, and CI in identifying a high cardiometabolic risk are shown in Table 2 and Figure 2. In men, when considering the full sample, the best cut-off vales of BMI, WtHR, BRI, ABSI, and CI for detecting high cardiometabolic risk (CMRI ≥ 1 SD) were 25.2 (area under curve, AUC 0.76, sensitivity 84.4% and specificity 54.7%), 0.56 (AUC 0.77, sensitivity 83.6% and specificity 58.9%), 4.71 (AUC 0.77, sensitivity 83.6% and specificity 58.9%), 0.083 (AUC 0.60, sensitivity 69.5% and specificity 53.6%), and 22.9 (AUC 0.75, sensitivity 72.3% and specificity 65.9%), respectively. For women, the best cut-off values of BMI, WtHR, BRI, ABSI, and CI for detecting high cardiometabolic risk (CMRI ≥ 1 SD) were 28.4 (AUC 0.71, sensitivity 69.5% and specificity 64.1%), 0.63 (AUC 0.77, sensitivity 64.4% and specificity 76.7%), 6.20 (AUC 0.77, sensitivity 65.2% and specificity 76.1%), 0.080 (AUC 0.62, sensitivity 68.7% and specificity 51.6%), and 21.0 (AUC 0.71, sensitivity 63.6% and specificity 70.2%), respectively.

The ROC curves were compared using a pairwise comparison method and the differences between the five methods are shown in Table 3. Independently of sex, the ROC-AUC of WtHR did not significantly differ from that of BRI. The results indicated that WtHR and BRI seem to provide the best results in Colombian older adults, owing to their greater precision in identifying subjects with a high cardiometabolic risk.

### 3.4. Sex Thresholds for Surrogate Anthropometric Indices to Screen for CMRI

Thresholds were determined for each of the surrogate anthropometric indices for the low/high CMRI in males and females, with corresponding differences in cardiometabolic parameters (Figure 2 and Table 4). In all groups (healthy/unhealthy) thresholds may be used to categorize individuals into one of two risk categories (i.e., low and high), on the combined basis of sex and surrogate anthropometric indices. In both sexes, after adjusting for ethnicity, socioeconomic status, smoking status, alcohol intake, physical activity proxy, and medical conditions (presence or absence of osteoporosis, CVD, hypertension, diabetes, cancer, and respiratory disease), the ANCOVA revealed that there were differences in blood pressure, HDL-C, and glucose in the BMI and CI parameters. By contrast, diagnostic performance results for CMRI without the central obesity component (i.e., WC) revealed lower accuracy (AUC) in all thresholds for surrogate anthropometric indices (Supplementary Material Table S1).

Finally, the collinearity test for all anthropometric indices that included WC in their calculation was found to be negative for BRI (VFI: 9.3), ABSI (VFI: 3.5), and WtHR (VFI: 9.1) and positive for CI (VFI: 10.9).

## 4. Discussion

Metabolic abnormalities including elevated blood pressure, hypertriglyceridemia, low levels of HDL-C, impaired glucose tolerance and central obesity, have been proposed as cardiometabolic risk factors for CVD and all-cause mortality [28,29]. For this reason, identifying a screening tool for detecting high cardiometabolic risk in older adults is particularly important, as this might facilitate the early implementation of effective strategies to those at high risk. This study investigated multiple anthropometric measurements for predicting cardiometabolic risk in a large population of older Colombian adults. Firstly, we demonstrated that all the surrogate anthropometric indices including BMI, WtHR, BRI, ABSI, and CI significantly correlated with CMRI. Secondly, we showed that WtHR and BRI are the most accurate anthropometric indices for identifying adults at high cardiometabolic risk, supporting the hypothesis that these two indices could effectively predict cardiometabolic risk in the elderly Colombian population.

In the present study, conducted on a representative cohort of older adults, the overall prevalence of MetS was 58.7% according to IDF criteria. These findings differ slightly from the results of Davila et al., who showed that the prevalence of MetS among adults from Medellin (Colombia) aged 25–64 was 41% [30]. Furthermore, the Cardiovascular Risk Factor Multiple Evaluation in Latin America (CARMELA) study estimated a prevalence of 30.1% in men and 48.6% in women, respectively, in the 55–64 age group in Bogotá [31]. The differences in prevalence could be explained by either the MetS cluster used, since the CARMELA study defined MetS according to the National Cholesterol Education Program Adult Treatment Panel III, or the age range of the target populations (55–64 vs. ≥60). Nonetheless, there is a high prevalence of MetS in Latin American populations and, accordingly, there is growing interest in developing accurate tools for identifying subjects at high risk and defining cut-off points for anthropometric indices for detecting high CMRI.

BRI is a novel body index that has recently shown promise for clinical use [15]. We found that BRI has a moderate discriminating power for detecting high cardiometabolic risk in older Colombian adults, supporting the diagnostic potential of this new shape measure. We found that BRI performed better as a predictor of a high CMRI than BMI, the standard measure. Similarly, Tian et al. observed that BRI was suitable for use as a single anthropometric measure for identifying a cluster of cardiometabolic abnormalities, as compared with BMI and WtHR, using data from the 2009 wave of the China Health and Nutrition Survey [32]. Likewise, a recent study assessing the ability of BRI to predict the risk of MetS and its components in Peruvian adults concluded that BRI is a potentially useful clinical predictor of MetS that performs better than BMI [18]. BRI also showed potential for use as an alternative obesity measure in type 2 diabetes mellitus assessment among a rural population from northeastern China, although it performed similarly to BMI [33]. Additionally, Maessen et al. found that BRI could identify both the presence of CVD and cardiovascular risk factors in a population-based study in Nijmegen, the Netherlands, although the authors indicated that its capacity did not exceed that of BMI [19]. The heterogeneity of the population characteristics (ethnicity and age range) might explain the differences between these studies.

We demonstrated that WtHR is also an accurate screening tool for detecting a high cardiometabolic risk in older Colombian adults. Indeed, we found that WtHR was a better predictor of cardiometabolic risk than other anthropometric indices (BMI, CI, and ABSI). Wang et al. [34] also indicated that when evaluating cardiometabolic risk factors among non-obese adults, WtHR functioned as a simple but effective index for Chinese adults and, similarly, Amirabdollahian et al. [35] concluded that WtHR was the best predictor of cardiometabolic risk in a population of young adults from northwestern England. Comparable results were reported in a previous systematic review and meta-analysis involving 300,000 adults from several ethnic groups [36], showing the superiority of WtHR over BMI for detecting cardiometabolic risk factors in both sexes. However, it should be noted that the aforementioned studies did not compare WtHR with ABSI or BRI.

Interestingly, it should be noted that the greatest AUC values were observed for WtHR and BRI in men and women, suggesting that both body indices are capable of detecting a high cardiometabolic risk in the elderly. In addition, the AUC of WtHR did not differ significantly from that of BRI. This highlights the similar diagnostic capabilities of the two anthropometric indices. Furthermore, the AUC value of BRI for identifying metabolic risk factors was very close to that of WtHR in a Chinese population of adults [37]. In fact, Wang et al. concluded that although BRI does not exhibit a significantly better predictive ability than WtHR, it could be used as an alternative body index [34].

Our results showed that ABSI presented the lowest AUC for high cardiometabolic risk in men and women. These observations are consistent with previous studies [18,19,20,35,37,38]. Tian et al. reported that ABSI had the weakest discriminative power for identifying a cluster of cardiometabolic abnormalities [32]. Similarly, ABSI exhibited the lowest AUC value for identifying cardiometabolic risk factors compared with WtHR and BRI in Chinese adults [37], and a study involving an Iranian population also reported that ABSI was a weak predictor of CVD risk and MetS [38]. In the same line, Stefanescu et al. found that ABSI underperformed against other measures such as BMI and BRI for predicting MetS and its components [18], and Maessen et al. reported that ABSI was incapable of determining the presence of CVD in a Dutch population [19]. Thus, based on both our results and those of previous research, it can be concluded that ABSI does not seem to be a useful anthropometric index for predicting cardiometabolic risk.

The present study has some limitations and strengths that should be mentioned. Firstly, the cross-sectional design of the study meant that causality could not be inferred. Secondly, all of the study participants were of Latin-American ethnicity and resident in Colombia. This may therefore limit the generalizability of our results to other ethnic groups. Further studies involving other populations are therefore warranted. By contrast, the main strength of our study is that we provide gender-specific thresholds for various surrogate anthropometric indices (BMI, WtHR, BRI, ABSI, and CI) with cardiometabolic measurements among older Colombian adults. To our knowledge, no research has previously been published assessing the efficacy of these anthropometric indices for predicting a high CMRI in a Latin-American population. Lastly, the large sample size and the highly standardized procedures of the SABE project, which minimized measurement bias, were also major strengths of this study [39,40].

## 5. Conclusions

In conclusion, BRI and WtHR have a moderate discriminating power for determining a high cardiometabolic risk in a Colombian population of older adults, supporting the notion that both anthropometric indices should be considered as screening tools for the elderly. Both anthropometric indices were the most accurate among those tested for identifying men and women at a high cardiometabolic risk. In addition, we provide the first BMI, WtHR, BRI, ABSI, and CI thresholds for predicting a high CRMI in older Colombian adults. These data are clinically significant, as anthropometric index reference thresholds can be used to identify those adults who are at high cardiometabolic risk. Further investigation is required to provide reference values applicable to different populations.

## Figures and Tables

**Table 1:** Characteristics of study participants according to high (≥ 1 SD) and low (< 1 SD) cardiometabolic risk index (CMRI) status among Colombian older adults.

Characteristics	Total Sample (*n* = 1502)	High CMRI ≥ 1 SD (*n* = 397)	Low CMRI < 1 SD (*n* = 1105)	*p*-Value
Sex, *n* (%)				
Men	596 (39.7)	141 (23.7)	455 (76.3)	**<0.001**
Women	906 (60.3)	254 (28.0)	652 (72.0)	**<0.001**
Socioeconomic status				
1	456 (30.4)	121 (30.5)	335 (32.1)	**<0.001**
2	635 (42.3)	176 (44.3)	459 (41.5)	**<0.001**
3	375 (25.0)	98 (24.7)	277 (25.1)	**<0.001**
4	29 (1.9)	2 (0.5)	27 (2.4)	**<0.001**
>5	7 (0.5)	0 (0.0)	7 (0.6)	N.A
Ethnic group				
Indigenous	78 (5.2)	25 (6.3)	53 (4.8)	**0.002**
Black	119 (7.9)	28 (7.1)	91 (8.2)	**<0.001**
White	396 (26.4)	106 (26.7)	290 (26.2)	**<0.001**
Others	909 (60.5)	194 (48.9)	512 (46.3)	**<0.001**
Smoking status, *n* (%)				
Yes	145 (9.7)	29 (7.3)	116 (10.5)	**<0.001**
No	1357 (90.3)	368 (92.7)	989 (89.5)	**<0.001**
Alcohol intake, *n* (%)				
Yes	191 (12.7)	52 (13.1)	139 (12.6)	**<0.001**
No	1310 (87.2)	345 (86.9)	965 (87.3)	**<0.001**
Physical Activity “proxy”, *n* (%)				
Physically active	266 (17.7)	70 (17.6)	196 (17.7)	0.980
Non-Physically active	1231 (82.0)	323 (81.4)	908 (82.2)	**<0.001**
Anthropometric measures/indices				
Height (m)	1.55 (1.49–1.62)	1.54 (1.49–1.62)	1.55 (1.49–1.62)	0.170
Weight (kg)	64 (57–72)	71 (63–79)	62 (55–69)	**<0.001**
Waist circumference (cm)	92 (85–100)	101 (93–107)	89 (83–97)	**<0.001**
Body mass index (kg/m^2^)	27 (24–30)	29.7 (26.7–33)	26.1 (23.3–29)	**<0.001**
WtHR	0.59 (0.1)	0.64 (0.06)	0.57 (0.06)	**<0.001**
BRI	5.2 (4.1–6.3)	6.4 (5.3–7.7)	4.8 (3.9–5.9)	**<0.001**
ABSI (m^11/6^ ∙ kg ^−2/3^)	0.081 (0.078–0.085)	0.083 (0.080–0.086)	0.081 (0.077–0.084)	**<0.001**
CI	22.2 (20.9–23.8)	21.1 (19.8–22.4)	22.6 (21.4–24.1)	**<0.001**
Metabolic syndrome components, *n* (%)				
Prevalence of MetS	811 (58.7)	308 (77.6)	503 (45.5)	**<0.001**
Abdominal obesity	1177 (78.4)	374 (94.2)	803 (72.7)	**<0.001**
Hypertension	790 (52.6)	304 (76.6)	486 (44.0)	**<0.001**
High levels of fasting glucose	465 (31.0)	220 (55.4)	245 (22.2)	**<0.001**
High levels of triglycerides	696 (46.3)	253 (63.7)	443 (40.1)	**<0.001**
Low levels of HDL-C	821 (54.7)	219 (55.2)	602 (54.5)	0.393
Cardiometabolic measurements				
SBP (mmHg)	130 (117–145)	142 (130–163)	126 (114–140)	**<0.001**
DBP (mmHg)	72 (65–79)	78 (72–86)	70 (64–77)	**<0.001**
MBP (mmHg)	92 (84–101)	100 (91–111)	89 (81–97)	**<0.001**
Total cholesterol (mg/dL)	193 (166–221)	202 (171–232)	190 (164–216)	**<0.001**
Triglycerides (mg/dL)	144 (105–192)	174 (134–252)	134 (101–180)	**<0.001**
LDL-C (mg/dL)	126 (102–149)	127 (103–152)	125 (102–147)	0.116
HDL-C (mg/dL)	43 (36–53)	43 (36–54)	44 (36–53)	0.740
Glucose (mg/dL)	94 (86–102)	102 (93–121)	91 (84–98)	**<0.001**
CMRI	−0.21 (−1.41–1.07)	2.00 (1.44–2.84)	−0.83 (−1.83–0.05)	**<0.001**
Self-report comorbid chronic diseases, *n* (%)				
Hypertension	826 (55.0)	249 (62.7)	577 (52.2)	**<0.001**
Diabetes	245 (16.3)	113 (28.5)	132 (11.9)	**<0.001**
Respiratory diseases	165 (11.0)	49 (12.3)	116 (10.5)	**<0.001**
Cardiovascular diseases	213 (14.2)	155 (39.0)	58 (5.2)	**<0.001**
Stroke	70 (4.7)	22 (5.5)	48 (4.3)	**<0.001**
Osteoporosis	184 (12.3)	66 (16.6)	118 (10.7)	**<0.001**
Cancer	80 (5.3)	56 (14.1)	24 (2.2)	**<0.001**
Hearing loss	360 (24.1)	89 (22.4)	271 (24.5)	**<0.001**
Vision loss	851 (56.7)	228 (57.4)	623 (56.4)	**<0.001**

Skewed continuous variables are reported as median and interquartile range (Q3-Q1), for non-skewed continuous variables mean values (standard deviations (SD)) are given, and categorical variables are reported as numbers and percentages in brackets. Significant between-sex differences (Student’s *t*-test, Wilcoxon rank-sum test or χ2). BMI: body mass index; WtHR: waist-to-height ratio; BRI: body roundness index; ABSI: a body shape index; CI: conicity index; LDL-C: low-density lipoprotein cholesterol; HDL-C: high-density lipoprotein cholesterol; CMRI: cardiometabolic risk index. *p*-values marked in bold are significant.

**Table 2:** Cut-off points, area under curve, sensitivity and specificity for BMI, WtHR, BRI, ABSI, and CI to detect high cardiometabolic risk (CMRI ≥ 1 SD) by sex.

Parameters	BMI		WtHR		BRI		ABSI		CI	
	Men	Women	Men	Women	Men	Women	Men	Women	Men	Women
Area under curve	0.76	0.71	0.77	0.77	0.77	0.77	0.60	0.62	0.75	0.71
*p*-value	<0.0001	<0.0001	<0.0001	<0.0001	<0.0001	<0.0001	<0.0001	<0.0001	<0.0001	<0.0001
Optimal cut-off	25.2	28.4	0.56	0.63	4.71	6.20	0.083	0.080	22.9	21.0
Youden index J	0.39	0.33	0.42	0.41	0.42	0.41	0.23	0.20	0.38	0.33
Sensitivity (%)	84.4	69.5	83.6	64.4	83.6	65.2	69.5	68.7	72.3	63.6
Specificity (%)	54.7	64.1	58.9	76.7	58.9	76.1	53.6	51.6	65.9	70.2
(+) Likelihood ratio	1.83	1.93	2.00	2.70	2.04	2.74	1.50	1.42	2.12	2.14
(–) Likelihood ratio	0.29	0.48	0.28	0.47	0.28	0.46	0.57	0.60	0.42	0.52

BMI: body mass index; WtHR: waist to height ratio; BRI: body roundness index; ABSI: a body shape index; CI: conicity index.

**Table 3:** Pairwise comparison for receiver operating characteristic (ROC) curves among Colombian older adults by sex.

Parameters	BMI–WtHR	BMI–BRI	BMI–ABSI	BMI–CI	WtHR–BRI	WtHR–ABSI	WtHR–CI	BRI–ABSI	BRI–CI	ABSI–CI
**Men**										
Diff. AUC	0.000	0.00	0.15	0.01	0.00	0.16	0.01	0.16	0.02	0.14
SE	0.01	0.01	0.03	0.00	0.00	0.02	0.01	0.02	0.01	0.03
*p*-value	0.542	0.540	**0.001**	0.220	0.090	**0.001**	0.100	**0.001**	0.090	**0.001**
**Women**										
Diff. AUC	0.06	0.06	0.08	0.00	0.00	0.15	0.06	0.15	0.06	0.08
SE	0.01	0.01	0.03	0.00	0.00	0.02	0.01	0.02	0.01	0.03
*p*-value	**0.001**	**0.001**	**0.001**	0.99	0.97	**0.001**	**0.001**	**0.001**	**0.001**	**0.001**

AUC: area under curve; SE: standard error; BMI: body mass index; WtHR: waist to height ratio; BRI: body roundness index; ABSI: a body shape index; CI: conicity index. P-values marked in bold are significant.

**Table 4:** Adjusted thresholds for surrogate anthropometric indices with cardiometabolic measurements among Colombian older adults by sex.

Variables	Cut-Off	BMI		WtHR		BRI		ABSI		CI	
		Mean (SE)	*p*-Value	Mean (SE)	*p*-Value	Mean (SE)	*p*-Value	Mean (SE)	*p*-Value	Mean (SE)	*p*-Value
**Men**											
SBP (mmHg)	healthy	130.1 (1.5)	**0.001**	131.7 (1.5)	0.107	131.5 (1.4)	**0.045**	134.7 (2.1)	0.477	131.3 (1.3)	0.005
	unhealthy	136.1 (1.3)		135.0 (1.3)		135.6 (1.4)		133.3 (1.1)		136.6 (1.5)	
DBP (mmHg)	healthy	72.3 (0.8)	**0.001**	73.8 (0.8)	0.250	73.7 (0.7)	0.131	76.1 (13.4)	0.176	73.1 (0.7)	**0.003**
	unhealthy	76.3 (0.7)		75.3 (0.7)		75.4 (0.7)		74.1 (0.6)		76.5 (0.8)	
MBP (mmHg)	healthy	91.5 (1.0)	**0.001**	93.1 (0.9)	0.148	92.9 (0.9)	0.064	95.5 (1.3)	0.282	92.4 (0.8)	**0.003**
	unhealthy	96.1 (0.8)		95.0 (0.8)		95.4 (0.9)		93.8 (0.7)		96.4 (0.9)	
Total cholesterol (mg/dL)	healthy	189.5 (2.0)	**0.011**	190.0 (2.5)	**0.010**	188.0 (2.4)	0.099	186.6 (3.5)	0.947	186.6 (2.2)	0.224
	unhealthy	181.8 (2.2)		181.2 (2.2)		182.4 (2.3)		184.7 (1.9)		183.1 (2.5)	
Triglycerides (mg/dL)	healthy	149.6 (5.7)	0.22	149.4 (5.6)	0.088	151.4 (5.3)	0.176	147.4 (7.8)	00.085	150.7 (4.9)	00.054
	unhealthy	162.7 (4.9)		163.2 (4.9)		162.5 (5.2)		160.0 (4.2)		165.4 (5.6)	
LDL-C (mg/dL)	healthy	123.5 (2.2)	0.055	123.7 (2.1)	0.058	122.5 (2.0)	0.211	119.8 (3.0)	0.511	121.3 (1.9)	0.467
	unhealthy	118.4 (1.8)		118.1 (1.9)		118.8 (1.9)		120.8 (1.6)		119.6 (2.1)	
HDL-C (mg/dL)	healthy	45.1 (0.7)	**0.001**	44.3 (0.7)	**0.001**	44.3 (12.4)	**0.001**	45.4 (1.0)	**0.001**	43.9 (0.6)	**0.001**
	unhealthy	39.5 (0.6)		39.6 (0.6)		39.3 (9.5)		40.8 (0.5)		39.2 (0.7)	
Glucose (mg/dL)	healthy	93.7 (1.5)	**0.005**	93.9 (1.5)	**0.009**	93.6 (1.4)	**0.002**	98.0 (2.1)	0.519	95.0 (1.3)	**0.028**
	unhealthy	99.1 (1.3)		99.0 (1.3)		99.8 (1.4)		96.4 (1.1)		99.0 (1.5)	
CMRI	healthy	−1.09 (0.1)	**0.001**	−1.05 (0.1)	**0.001**	−1.05 (0.1)	**0.001**	−0.66 (0.18)	**0.010**	−0.87 (0.11)	**0.001**
	unhealthy	0.36 (0.1)		0.36 (0.1)		0.50 (0.1)		−0.13 (0.09)		0.53 (0.12)	
**Women**											
SBP (mmHg)	healthy	130.4 (1.1)	0.530	130.6 (1.0)	0.942	130.6 (1.0)	0.935	129.8 (1.2)	0.639	130.4 (1.0)	0.537
	unhealthy	131.1 (1.1)		130.8 (1.3)		130.8 (1.3)		131.4 (1.0)		131.1 (1.2)	
DBP (mmHg)	healthy	70.9 (0.5)	**0.021**	71.6 (0.5)	0.458	71.6 (0.5)	0.414	72.0 (0.6)	0.993	71.1 (0.5)	**0.034**
	unhealthy	72.9 (10.5)		72.3 (0.6)		72.3 (0.6)		71.8 (0.5)		73.1 (0.6)	
MBP (mmHg)	healthy	90.6 (0.6)	0.097	91.2 (0.6)	0.721	91.2 (0.6)	0.691	91.2 (0.7)	0.823	90.8 (0.6)	0.139
	unhealthy	92.3 (0.7)		91.7 (0.8)		91.8 (0.8)		91.6 (0.6)		92.4 (0.7)	
Total cholesterol (mg/dL)	healthy	203.7 (1.9)	0.233	204.1 (1.8)	0.098	203.9 (1.8)	0.149	202.0 (2.1)	0.572	203.2 (1.8)	0.376
	unhealthy	200.4 (2.1)		198.8 (2.3)		199.0 (2.4)		202.2 (1.9)		200.5 (2.2)	
Triglycerides (mg/dL)	healthy	161.1 (4.0)	0.111	160.8 (3.7)	0.059	161.2 (3.7)	0.068	157.0 (4.4)	**0.044**	162.3 (3.8)	0.159
	unhealthy	170.8 (4.3)		173.5 (4.8)		173.4 (4.9)		172.4 (3.9)		170.7 (4.7)	
LDL-C (mg/dL)	healthy	132.3 (1.7)	0.258	132.6 (1.6)	0.164	132.5 (1.5)	0.132	132.0 (34.8)	0.812	132.0 (1.6)	0.381
	unhealthy	129.8 (1.8)		128.8 (2.0)		128.8 (2.1)		131.1 (37.4)		129.9 (2.0)	
HDL-C (mg/dL)	healthy	48.8 (0.6)	**0.009**	48.8 (0.5)	**0.001**	48.6 (0.5)	**0.007**	48.7 (13.9)	0.104	48.5 (0.5)	**0.018**
	unhealthy	46.1 (0.6)		45.4 (0.7)		45.6 (0.7)		46.9 (12.3)		46.1 (0.7)	
Glucose (mg/dL)	healthy	97.3 (1.1)	**0.016**	96.8 (1.0)	**0.001**	96.6 (1.0)	**0.001**	98.1 (1.2)	0.241	97.0 (1.0)	**0.002**
	unhealthy	101.2 (1.2)		102.9 (1.3)		103.5 (1.3)		99.9 (1.1)		102.3 (1.3)	
CMRI	healthy	−0.61 (0.09)	**0.001**	−0.57 (0.08)	**0.001**	−0.56 (0.08)	**0.001**	−0.32 (0.10)	**0.001**	−0.52 (0.08)	**0.001**
	unhealthy	0.82 (0.09)		1.09 (0.10)		1.16 (0.01)		0.36 (0.09)		0.94 (0.10)	

Data reported as mean and standard error (SE). BMI: body mass index; WtHR: waist-to-height ratio; BRI: body roundness index; ABSI: a body shape index; CI: conicity index; SBP: systolic blood pressure; DBP: diastolic blood pressure; MBP: mean blood pressure. *p*-value from ANCOVA analysis performed with ethnicity, socio-economic status, smoking status, alcohol intake, physical activity “proxy”, and medical conditions (i.e., presence or absence of osteoporosis, cardiovascular diseases, hypertension, diabetes, cancer, or respiratory disease) as covariates. *p*-values marked in bold are significant.

## Supplementary Materials

The following are available online at https://www.mdpi.com/2072-6643/11/8/1701/s1, Table S1: Area under curve for BMI, WHTR, BRI, ABSI, and CI to detect cardiometabolic risk (without WC) by sex.
