# Correction: Ramírez-Vélez, R.; et al. Validation of Surrogate Anthropometric Indices in Older Adults: What Is the Best Indicator of High Cardiometabolic Risk Factor Clustering? *Nutrients* 2019, *11*, 1701

**DOI:** 10.3390/nu11102413

**Published:** 2019-10-10

**Authors:** Robinson Ramírez-Vélez, Miguel Ángel Pérez-Sousa, Mikel Izquierdo, Carlos A. Cano-Gutierrez, Emilio González-Jiménez, Jacqueline Schmidt-RioValle, Katherine González-Ruíz, María Correa-Rodríguez

**Affiliations:** 1 Department of Health Sciences, Public University of Navarra, Navarrabiomed-Biomedical Research Centre, IDISNA-Navarra’s Health Research Institute, C/irunlarrea 3, Complejo Hospitalario de Navarra, 31008 Pamplona, Navarra, Spain; mikel.izquierdo@gmail.com; 2 Faculty of Sport Sciences, University of Huelva, Avenida de las Fuerzas Armadas s/n 21007, 21004 Huelva, Spain; perezsousa@gmail.com; 3 Centro de Investigación Biomédica en Red de Fragilidad y Envejecimiento Saludable (CIBERFES), Instituto de Salud Carlos III, 28029 Madrid, Spain; 4 Hospital Universitario San Ignacio—Aging Institute, Pontificia Universidad Javeriana, Bogotá 110111, Colombia; ccano@javeriana.edu.co; 5 Department of Nursing, Faculty of Health Sciences, University of Granada, Av. Ilustración, 60, 18016 Granada, Spain; emigoji@ugr.es (E.G.-J.); macoro@ugr.es (M.C.-R.); 6 Grupo de Ejercicio Físico y Deportes, Vicerrectoría de Investigaciones, Facultad de Salud, Universidad Manuela Beltrán, Bogotá 110231, Colombia; katherine.gonzalez@docentes.umb.edu.co

The authors would like to make the following corrections to the published paper [1]:

(1) In the result section replace:
  On page 6, in the text of the Section 3.1, ‘22.2 (20.9–23.8),’ must be replaced by ‘1.31 (1.26–1.37).’
  On page 7, in the text of the Section 3.2, (*r* = −0.42, *p* < 0.001)’, ’CI (*r* = −0.46, *p* < 0.001)’ and ’CI (*r* = −0.44, *p* < 0.001)’, must be replaced by (*r* = 0.38, *p* < 0.001)’, ’CI (*r* = 0.31, *p* < 0.001)’ and ’CI (*r* = 0.41, *p* < 0.001)’.
  On page 9, in the text of the Section 3.3, ‘22.9’and ‘21.0,’ must be replaced by ‘1.33’and ’1.31’.
  On page 10, in the text of the Section 3.4, ‘10.9,’ must be replaced by ’12.8’.
(2) In Table 1 replace:
  On page 6, Section 3.1, Table 1, ’22.2 (20.9–23.8)’, ’22.2 (20.9–23.8)’ and ’22.2 (20.9–23.8)’, must be replaced by ’1.31 (1.26–1.37)’, ’1.34 (1.29–1.39)’, ’1.30 (1.24–1.35)’.
(3) In Figure 1 replace:
  On page 8, Section 3.2, Figure 1, Panel CI-CMRI, must be replaced by:
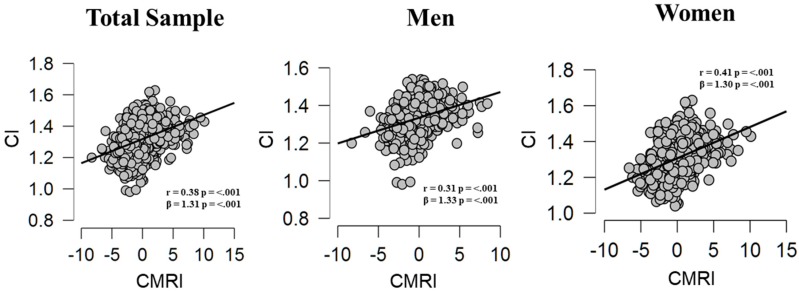
(4) In Table 2 replace:
  On page 9, Section 3.3, Table 2, ’22.9’ and ’21.0’, must be replaced by ’1.33’, and ’1.30’.
  These changes have no material impact on the conclusions of our paper. The manuscript will be updated and the original will remain online on the article webpage. The authors apologize for these errors and state that this does not change the scientific conclusions of the article in any way.
