# Supplementary material for: Validation of Surrogate Anthropometric Indices in Older Adults: What Is the Best Indicator of High Cardiometabolic Risk Factor Clustering?
Source: Nutrients. 2019 Jul 24;11(8):1701. doi: 10.3390/nu11081701 (PMC6723899; doi:10.3390/nu11081701)
Supplement: Supplementary file 1 [file nutrients-11-01701-s001.pdf]

Supplementary Table 1.

Area under curve for BMI, WHTR, BRI, ABSI and CI to detect cardiometabolic risk (without waist circumference) by sex.

	BMI		WHtR		BRI		ABSI		C-Index	
	Men	Women	Men	Women	Men	Women	Men	Women	Men	Women
**Area under curve**	0.55	0.52	0.54	0.53	0.54	0.53	0.50	0.52	0.55	0.52
**P-value**	0.08	0.29	0.21	0.09	0.21	0.09	0.96	0.36	0.09	0.25
**Optimal cut-off**	25.7	30.3	0.57	0.59	4.77	5.26	0.083	0.08	21.2	20.1
**Youden index J**	0.13	0.05	0.09	0.08	0.10	0.08	0.10	0.05	0.09	0.07
**Sensitivity (%)**	0.00	34.56	57.29	63.13	57.29	63.13	60.42	26.73	19.79	28.57
**Specificity (%)**	100.0	71.32	52.60	45.05	52.80	44.98	49.80	78.60	90.20	78.72
**(+) Likelihood ratio**	1.28	1.19	1.17	1.14	1.21	1.15	1.16	1.19	1.41	1.34
**(–) Likelihood ratio**	0.74	0.92	0.86	0.82	0.81	0.82	0.84	0.94	0.89	0.91

BMI: Body Mass Index; WHtR: Waist-to-Height Ratio; BRI: Body Roundness Index; ABSI: A Body Shape Index; C-Index: Conicity Index.
